# Paraherquamide E

**DOI:** 10.1107/S1600536810030795

**Published:** 2010-08-11

**Authors:** Thammarat Aree, Bassey S. Antia, Okon D. Ekpa, Prasat Kittakoop

**Affiliations:** a Department of Chemistry, Faculty of Science, Chulalongkorn University, Phyathai Road, Pathumwan, Bangkok 10330, Thailand; b Department of Pure and Applied Chemistry, University of Calabar, Calabar, Nigeria; c Chulabhorn Research Institute and Chulabhorn Graduate Institute, Chemical Biology Program, Vibhavadi-Rangsit Highway, Bangkok 10210, Thailand

## Abstract

In the title compound, C_28_H_35_N_3_O_4_, also known as 14-de­oxy­paraherquamide A,the two pyrrolidine rings adopt envelope conformations. The piperazine ring of the diaza­bicyclo­[2.2.2]octan-3-one unit adopts a boat conformation whereas the two piperidine rings are in distorted boat conformations. Intra­molecular C—H⋯O hydrogen bonds are observed. In the crystal, the mol­ecules are linked into chains along the *b* axis by inter­molecular N—H⋯O hydrogen bonds.

## Related literature

For the structure determination of paraherquamides, see: Liesch & Wichmann (1990 ▶). For the crystal structure of paraherquamide A, see: Yamazaki *et al.* (1981 ▶). For the anti­nematodal and anti­parasitic activities of paraherquamides, see: Ondeyka *et al.* (1990 ▶). For their anthelmintic activity, see: Blanchflower *et al.* (1991 ▶) and for their insecticidal activity, see: Lopez-Gresa *et al.* (2006 ▶). For reviews on the total synthesis and biosynthesis of paraherquamides, see: Williams (2002 ▶); Williams & Cox (2003 ▶).
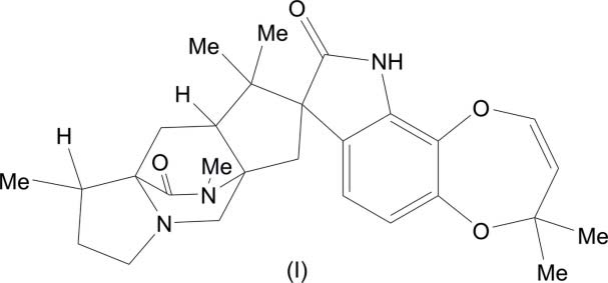


## Experimental

### 

#### Crystal data

C_28_H_35_N_3_O_4_
*M* *_r_* = 477.59
Orthorhombic, 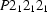

*a* = 6.5069 (2) Å
*b* = 16.0351 (8) Å
*c* = 23.9713 (11) Å
*V* = 2501.14 (19) Å^3^
*Z* = 4
Mo *K*α radiation
μ = 0.09 mm^−1^
*T* = 298 K
0.40 × 0.10 × 0.10 mm

#### Data collection

Bruker SMART APEXII CCD area-detector diffractometer
Absorption correction: multi-scan (*SADABS*; Bruker, 2005 ▶) *T*_min_ = 0.946, *T*_max_ = 0.954
8444 measured reflections
2889 independent reflections
1746 reflections with *I* > 2σ(*I*)
*R*_int_ = 0.049

#### Refinement

*R*[*F* ^2^ > 2σ(*F* ^2^)] = 0.049
*wR*(*F* ^2^) = 0.102
*S* = 0.98
2889 reflections
322 parameters
H-atom parameters constrained
Δρ_max_ = 0.16 e Å^−3^
Δρ_min_ = −0.20 e Å^−3^

### 

Data collection: *APEX2* (Bruker, 2005 ▶); cell refinement: *SAINT* (Bruker, 2005 ▶); data reduction: *SAINT*; program(s) used to solve structure: *SHELXTL* (Sheldrick, 2008 ▶); program(s) used to refine structure: *SHELXTL*; molecular graphics: *Mercury* (Macrae *et al.* 2006 ▶); software used to prepare material for publication: *SHELXTL*.

## Supplementary Material

Crystal structure: contains datablocks I, global. DOI: 10.1107/S1600536810030795/ci5149sup1.cif

Structure factors: contains datablocks I. DOI: 10.1107/S1600536810030795/ci5149Isup2.hkl

Additional supplementary materials:  crystallographic information; 3D view; checkCIF report

## Figures and Tables

**Table 1:** Hydrogen-bond geometry (Å, °)

*D*—H⋯*A*	*D*—H	H⋯*A*	*D*⋯*A*	*D*—H⋯*A*
N1—H1⋯O4^i^	0.86	2.21	2.968 (4)	147
C17—H17*A*⋯O4	0.96	2.39	3.016 (5)	123
C20—H20⋯O1	0.98	2.44	3.126 (4)	126

Symmetry code: (i) 
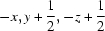
.

**Fig. 1.**
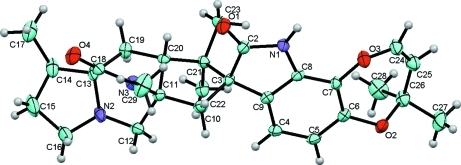
The molecular structure of the title compound, with atomic numbering scheme and 30% probability displacement ellipsoids.

**Fig. 2.**
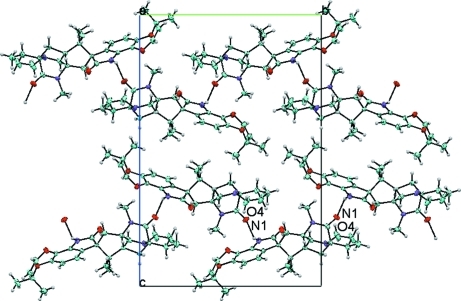
A view of the N—H···O hydrogen-bonded (dotted lines) chains of paraherquamide molecules along the b axis.

## supplementary crystallographic information

### Comment

The title compound, paraherquamide E (Fig. 1), was isolated from the marine-derived fungus *Aspergillus aculeatus*. In the family of the paraherquamides A–I, only the crystal structure of paraherquamide A has been reported thus far (Yamazaki *et al.*, 1981). We report here the crystal structure of paraherquamide E.

The molecular structure of the title compound comprises one diazabicyclo[2.2.2]octan-3-one unit, one cyclopentane ring, one 1,4-dioxepine ring and two pyrrolidine rings one in the middle and the other in the left end of the molecule. The two pyrrolidine rings adopt envelope conformations with atoms C3 and C13 as flaps. The piperazine ring of the diazabicyclo[2.2.2]octan-3-one unit adopts a boat conformation whereas the two piperidine rings are in distorted boat conformations.

The molecular structures of paraherquamide A and E are very similar and can be superimposed with r.m.s. deviation of superposition 0.15 Å (all H-atoms are excluded from the calculation).

In the crystal, the molecules are linked into chains along the *b*-axis by intermolecular N1—H···O4 hydrogen bonds (Table 1 and Fig. 2).

### Experimental

The title compound was isolated from the marine-derived fungus *Aspergillus aculeatus* and the single crystals were obtained by slow evaporation of a methanol–hexane (9:1, *v*/*v*) solution at room temperature.

### Refinement

All H atoms were located in a difference Fourier map and then refined using a riding model: C–H = 0.98 Å (tertiary), 0.97 Å(secondary), 0.93 Å (aromatic), 0.96 Å (methyl), N–H = 0.86 Å (amide), and *U*_iso_(H) = 1.2*U*_eq_(C,N) and *U*_iso_(H) = 1.5*U*_eq_(methyl C). In the absence of significant anomalous scattering effects, Friedel pairs were averaged.

### Crystal data

**Table:**

C_28_H_35_N_3_O_4_	*D*_x_ = 1.268 Mg m^−^^3^
*M**_r_* = 477.59	Melting point: not measured K
Orthorhombic, *P*2_1_2_1_2_1_	Mo *K*α radiation, λ = 0.71073 Å
Hall symbol: P 2ac 2ab	Cell parameters from 1372 reflections
*a* = 6.5069 (2) Å	θ = 2.5–19.7°
*b* = 16.0351 (8) Å	µ = 0.09 mm^−^^1^
*c* = 23.9713 (11) Å	*T* = 298 K
*V* = 2501.14 (19) Å^3^	Needle, colourless
*Z* = 4	0.40 × 0.10 × 0.10 mm
*F*(000) = 1024	

### Data collection

**Table:**

Bruker SMART APEXII CCD area-detector diffractometer	2889 independent reflections
Radiation source: fine-focus sealed tube	1746 reflections with *I* > 2σ(*I*)
graphite	*R*_int_ = 0.049
φ and ω scans	θ_max_ = 28.2°, θ_min_ = 1.5°
Absorption correction: multi-scan (*SADABS*; Bruker, 2005)	*h* = −5→8
*T*_min_ = 0.946, *T*_max_ = 0.954	*k* = −20→15
8444 measured reflections	*l* = −31→26

### Refinement

**Table:**

Refinement on *F*^2^	Primary atom site location: structure-invariant direct methods
Least-squares matrix: full	Secondary atom site location: difference Fourier map
*R*[*F*^2^ > 2σ(*F*^2^)] = 0.049	Hydrogen site location: inferred from neighbouring sites
*wR*(*F*^2^) = 0.102	H-atom parameters constrained
*S* = 0.98	*w* = 1/[σ^2^(*F*_o_^2^) + (0.0463*P*)^2^] where *P* = (*F*_o_^2^ + 2*F*_c_^2^)/3
2889 reflections	(Δ/σ)_max_ = 0.001
322 parameters	Δρ_max_ = 0.16 e Å^−^^3^
0 restraints	Δρ_min_ = −0.20 e Å^−^^3^

### Special details

**Table:**

Geometry. All e.s.d.'s (except the e.s.d. in the dihedral angle between two l.s. planes) are estimated using the full covariance matrix. The cell e.s.d.'s are taken into account individually in the estimation of e.s.d.'s in distances, angles and torsion angles; correlations between e.s.d.'s in cell parameters are only used when they are defined by crystal symmetry. An approximate (isotropic) treatment of cell e.s.d.'s is used for estimating e.s.d.'s involving l.s. planes.
Refinement. Refinement of *F*^2^ against ALL reflections. The weighted *R*-factor *wR* and goodness of fit *S* are based on *F*^2^, conventional *R*-factors *R* are based on *F*, with *F* set to zero for negative *F*^2^. The threshold expression of *F*^2^ > σ(*F*^2^) is used only for calculating *R*-factors(gt) *etc*. and is not relevant to the choice of reflections for refinement. *R*-factors based on *F*^2^ are statistically about twice as large as those based on *F*, and *R*- factors based on ALL data will be even larger.

### Fractional atomic coordinates and isotropic or equivalent isotropic displacement parameters (Å^2^)

**Table:**

	*x*	*y*	*z*	*U*_iso_*/*U*_eq_	
N1	0.0595 (4)	0.84401 (16)	0.16792 (11)	0.0366 (7)	
H1	−0.0514	0.8699	0.1769	0.044*	
N2	0.6009 (5)	0.47354 (18)	0.15605 (13)	0.0452 (8)	
N3	0.3520 (4)	0.54738 (18)	0.23432 (11)	0.0353 (7)	
O1	−0.0133 (4)	0.71770 (14)	0.20826 (10)	0.0510 (7)	
O2	0.4573 (4)	1.06380 (14)	0.07563 (9)	0.0421 (7)	
O3	0.0828 (4)	1.01425 (14)	0.13216 (10)	0.0468 (7)	
O4	0.2499 (4)	0.41513 (15)	0.25453 (10)	0.0543 (7)	
C2	0.0988 (6)	0.7623 (2)	0.17997 (14)	0.0364 (9)	
C3	0.3025 (5)	0.73668 (19)	0.15236 (13)	0.0293 (8)	
C4	0.5582 (5)	0.8436 (2)	0.10529 (14)	0.0387 (9)	
H4	0.6645	0.8054	0.1007	0.046*	
C5	0.5796 (5)	0.9252 (2)	0.08689 (14)	0.0396 (9)	
H5	0.7014	0.9414	0.0697	0.047*	
C6	0.4235 (5)	0.9831 (2)	0.09359 (13)	0.0336 (8)	
C7	0.2432 (6)	0.9606 (2)	0.12015 (14)	0.0327 (8)	
C8	0.2239 (5)	0.8798 (2)	0.13901 (14)	0.0314 (8)	
C9	0.3764 (5)	0.8200 (2)	0.13052 (13)	0.0321 (8)	
C10	0.4413 (6)	0.6932 (2)	0.19609 (14)	0.0393 (9)	
H10A	0.5828	0.7110	0.1916	0.047*	
H10B	0.3968	0.7074	0.2335	0.047*	
C11	0.4239 (5)	0.5991 (2)	0.18678 (12)	0.0308 (8)	
C12	0.6319 (5)	0.5608 (2)	0.17134 (15)	0.0434 (10)	
H12A	0.6918	0.5910	0.1403	0.052*	
H12B	0.7252	0.5646	0.2028	0.052*	
C13	0.3851 (5)	0.4474 (2)	0.16074 (14)	0.0353 (9)	
C14	0.4053 (6)	0.3551 (2)	0.14645 (17)	0.0553 (11)	
H14	0.4357	0.3520	0.1065	0.066*	
C15	0.6035 (8)	0.3309 (3)	0.1771 (2)	0.0952 (18)	
H15A	0.5717	0.3047	0.2126	0.114*	
H15B	0.6839	0.2923	0.1549	0.114*	
C16	0.7211 (7)	0.4117 (3)	0.1863 (2)	0.0831 (16)	
H16A	0.7289	0.4254	0.2256	0.100*	
H16B	0.8593	0.4078	0.1713	0.100*	
C17	0.2264 (8)	0.2967 (2)	0.15698 (19)	0.0720 (14)	
H17A	0.1929	0.2970	0.1960	0.108*	
H17B	0.2634	0.2412	0.1458	0.108*	
H17C	0.1094	0.3149	0.1358	0.108*	
C18	0.3167 (5)	0.4666 (2)	0.22117 (14)	0.0377 (9)	
C19	0.2626 (6)	0.50045 (19)	0.11950 (13)	0.0371 (9)	
H19A	0.1244	0.4786	0.1156	0.045*	
H19B	0.3282	0.4998	0.0832	0.045*	
C20	0.2558 (5)	0.58959 (18)	0.14247 (12)	0.0282 (8)	
H20	0.1259	0.5933	0.1630	0.034*	
C21	0.2623 (5)	0.66842 (19)	0.10578 (13)	0.0311 (8)	
C22	0.4342 (6)	0.6665 (2)	0.06154 (14)	0.0441 (10)	
H22A	0.5655	0.6642	0.0798	0.066*	
H22B	0.4266	0.7159	0.0390	0.066*	
H22C	0.4175	0.6182	0.0383	0.066*	
C23	0.0595 (6)	0.6830 (2)	0.07491 (15)	0.0463 (10)	
H23A	0.0437	0.6417	0.0462	0.069*	
H23B	0.0603	0.7375	0.0584	0.069*	
H23C	−0.0527	0.6788	0.1008	0.069*	
C24	0.0153 (6)	1.0726 (2)	0.09430 (16)	0.0498 (10)	
H24	−0.1173	1.0923	0.1003	0.060*	
C25	0.1105 (6)	1.1046 (2)	0.05100 (17)	0.0523 (11)	
H25	0.0318	1.1411	0.0298	0.063*	
C26	0.3240 (6)	1.0919 (2)	0.03039 (14)	0.0456 (10)	
C27	0.4195 (8)	1.1744 (2)	0.0128 (2)	0.0788 (15)	
H27A	0.4292	1.2107	0.0446	0.118*	
H27B	0.3351	1.2001	−0.0152	0.118*	
H27C	0.5543	1.1646	−0.0021	0.118*	
C28	0.3285 (7)	1.0286 (3)	−0.01689 (16)	0.0696 (14)	
H28A	0.4682	1.0189	−0.0280	0.104*	
H28B	0.2518	1.0497	−0.0480	0.104*	
H28C	0.2685	0.9771	−0.0044	0.104*	
C29	0.3185 (8)	0.5797 (3)	0.28994 (15)	0.0709 (14)	
H29A	0.2896	0.5344	0.3149	0.106*	
H29B	0.2042	0.6175	0.2895	0.106*	
H29C	0.4394	0.6086	0.3023	0.106*	

### Atomic displacement parameters (Å^2^)

**Table:**

	*U*^11^	*U*^22^	*U*^33^	*U*^12^	*U*^13^	*U*^23^
N1	0.0304 (16)	0.0298 (18)	0.0497 (17)	0.0040 (13)	0.0168 (14)	0.0009 (14)
N2	0.0426 (19)	0.0366 (19)	0.056 (2)	0.0087 (16)	0.0165 (16)	0.0203 (16)
N3	0.0423 (18)	0.0410 (19)	0.0227 (14)	−0.0021 (14)	0.0018 (13)	0.0055 (13)
O1	0.0489 (17)	0.0379 (16)	0.0663 (17)	−0.0022 (12)	0.0272 (14)	0.0053 (13)
O2	0.0468 (16)	0.0336 (15)	0.0457 (15)	−0.0107 (12)	−0.0008 (13)	0.0057 (12)
O3	0.0510 (16)	0.0383 (15)	0.0513 (15)	0.0103 (13)	0.0191 (13)	0.0106 (13)
O4	0.0655 (18)	0.0513 (16)	0.0460 (16)	−0.0100 (15)	0.0173 (14)	0.0181 (14)
C2	0.037 (2)	0.032 (2)	0.040 (2)	−0.0045 (18)	0.0061 (18)	−0.0024 (17)
C3	0.0302 (19)	0.0281 (19)	0.0296 (18)	−0.0024 (15)	0.0045 (16)	0.0010 (15)
C4	0.027 (2)	0.037 (2)	0.052 (2)	−0.0009 (16)	0.0052 (18)	−0.0025 (18)
C5	0.027 (2)	0.042 (2)	0.050 (2)	−0.0092 (19)	0.0052 (18)	−0.0007 (18)
C6	0.037 (2)	0.029 (2)	0.0343 (19)	−0.0059 (18)	0.0019 (18)	0.0009 (16)
C7	0.036 (2)	0.025 (2)	0.0365 (19)	0.0032 (17)	0.0030 (17)	−0.0035 (15)
C8	0.0300 (19)	0.031 (2)	0.0329 (19)	−0.0055 (16)	0.0043 (16)	−0.0063 (16)
C9	0.032 (2)	0.032 (2)	0.0316 (18)	−0.0039 (17)	0.0001 (17)	−0.0014 (16)
C10	0.035 (2)	0.043 (2)	0.040 (2)	−0.0051 (17)	−0.0061 (17)	−0.0005 (17)
C11	0.0298 (19)	0.035 (2)	0.0275 (17)	−0.0047 (16)	−0.0012 (16)	0.0076 (15)
C12	0.029 (2)	0.051 (3)	0.050 (2)	0.0013 (17)	−0.0004 (18)	0.0189 (19)
C13	0.039 (2)	0.029 (2)	0.038 (2)	0.0025 (16)	0.0131 (18)	0.0098 (17)
C14	0.069 (3)	0.034 (2)	0.063 (3)	0.010 (2)	0.024 (2)	0.011 (2)
C15	0.083 (4)	0.055 (3)	0.148 (5)	0.026 (3)	0.021 (4)	0.048 (3)
C16	0.056 (3)	0.065 (3)	0.129 (4)	0.022 (3)	0.012 (3)	0.046 (3)
C17	0.093 (4)	0.040 (2)	0.083 (3)	−0.002 (2)	0.023 (3)	0.002 (2)
C18	0.032 (2)	0.042 (2)	0.040 (2)	0.0033 (17)	0.0061 (17)	0.0121 (19)
C19	0.052 (2)	0.031 (2)	0.0282 (18)	0.0012 (18)	0.0014 (18)	0.0000 (16)
C20	0.0256 (17)	0.0321 (19)	0.0269 (18)	−0.0008 (15)	0.0028 (15)	0.0017 (15)
C21	0.0311 (19)	0.035 (2)	0.0272 (18)	−0.0002 (16)	−0.0017 (16)	0.0050 (15)
C22	0.055 (3)	0.041 (2)	0.036 (2)	0.005 (2)	0.0101 (19)	0.0052 (17)
C23	0.046 (2)	0.043 (2)	0.050 (2)	0.000 (2)	−0.0187 (19)	0.0070 (18)
C24	0.047 (2)	0.038 (2)	0.065 (3)	0.0089 (19)	0.015 (2)	0.007 (2)
C25	0.053 (3)	0.049 (3)	0.055 (2)	0.008 (2)	0.005 (2)	0.013 (2)
C26	0.051 (2)	0.045 (2)	0.040 (2)	0.0019 (19)	0.010 (2)	0.0102 (19)
C27	0.084 (4)	0.052 (3)	0.100 (4)	−0.003 (3)	0.019 (3)	0.037 (3)
C28	0.081 (4)	0.087 (3)	0.041 (2)	0.016 (3)	0.003 (2)	−0.003 (2)
C29	0.120 (4)	0.064 (3)	0.029 (2)	−0.019 (3)	0.012 (2)	0.004 (2)

### Geometric parameters (Å, °)

**Table:**

N1—C2	1.366 (4)	C14—C15	1.534 (6)
N1—C8	1.398 (4)	C14—H14	0.98
N1—H1	0.86	C15—C16	1.521 (7)
N2—C16	1.456 (5)	C15—H15A	0.97
N2—C12	1.460 (4)	C15—H15B	0.97
N2—C13	1.470 (5)	C16—H16A	0.97
N3—C18	1.353 (4)	C16—H16B	0.97
N3—C29	1.447 (4)	C17—H17A	0.96
N3—C11	1.485 (4)	C17—H17B	0.96
O1—C2	1.226 (4)	C17—H17C	0.96
O2—C6	1.382 (4)	C19—C20	1.532 (4)
O2—C26	1.460 (4)	C19—H19A	0.97
O3—C24	1.376 (4)	C19—H19B	0.97
O3—C7	1.383 (4)	C20—C21	1.541 (4)
O4—C18	1.228 (4)	C20—H20	0.98
C2—C3	1.537 (5)	C21—C23	1.531 (5)
C3—C9	1.513 (4)	C21—C22	1.541 (5)
C3—C10	1.550 (4)	C22—H22A	0.96
C3—C21	1.585 (4)	C22—H22B	0.96
C4—C9	1.382 (5)	C22—H22C	0.96
C4—C5	1.388 (4)	C23—H23A	0.96
C4—H4	0.93	C23—H23B	0.96
C5—C6	1.385 (5)	C23—H23C	0.96
C5—H5	0.93	C24—C25	1.313 (5)
C6—C7	1.382 (5)	C24—H24	0.93
C7—C8	1.378 (4)	C25—C26	1.489 (5)
C8—C9	1.395 (5)	C25—H25	0.93
C10—C11	1.529 (5)	C26—C28	1.522 (5)
C10—H10A	0.97	C26—C27	1.522 (5)
C10—H10B	0.97	C27—H27A	0.96
C11—C12	1.532 (5)	C27—H27B	0.96
C11—C20	1.533 (4)	C27—H27C	0.96
C12—H12A	0.97	C28—H28A	0.96
C12—H12B	0.97	C28—H28B	0.96
C13—C14	1.525 (5)	C28—H28C	0.96
C13—C19	1.528 (5)	C29—H29A	0.96
C13—C18	1.546 (5)	C29—H29B	0.96
C14—C17	1.516 (6)	C29—H29C	0.96
			
C2—N1—C8	110.8 (3)	N2—C16—H16A	111.0
C2—N1—H1	124.6	C15—C16—H16A	111.0
C8—N1—H1	124.6	N2—C16—H16B	111.0
C16—N2—C12	117.0 (3)	C15—C16—H16B	111.0
C16—N2—C13	106.3 (3)	H16A—C16—H16B	109.0
C12—N2—C13	112.7 (3)	C14—C17—H17A	109.5
C18—N3—C29	122.1 (3)	C14—C17—H17B	109.5
C18—N3—C11	114.2 (3)	H17A—C17—H17B	109.5
C29—N3—C11	123.7 (3)	C14—C17—H17C	109.5
C6—O2—C26	115.2 (3)	H17A—C17—H17C	109.5
C24—O3—C7	121.8 (3)	H17B—C17—H17C	109.5
O1—C2—N1	124.4 (3)	O4—C18—N3	123.5 (3)
O1—C2—C3	126.5 (3)	O4—C18—C13	125.3 (3)
N1—C2—C3	109.1 (3)	N3—C18—C13	111.1 (3)
C9—C3—C2	100.8 (3)	C13—C19—C20	107.6 (3)
C9—C3—C10	116.5 (3)	C13—C19—H19A	110.2
C2—C3—C10	109.4 (3)	C20—C19—H19A	110.2
C9—C3—C21	114.7 (2)	C13—C19—H19B	110.2
C2—C3—C21	110.2 (3)	C20—C19—H19B	110.2
C10—C3—C21	105.2 (2)	H19A—C19—H19B	108.5
C9—C4—C5	118.9 (3)	C19—C20—C11	108.8 (3)
C9—C4—H4	120.6	C19—C20—C21	124.0 (3)
C5—C4—H4	120.6	C11—C20—C21	107.1 (2)
C6—C5—C4	121.4 (3)	C19—C20—H20	105.2
C6—C5—H5	119.3	C11—C20—H20	105.2
C4—C5—H5	119.3	C21—C20—H20	105.2
O2—C6—C7	121.5 (3)	C23—C21—C20	112.2 (3)
O2—C6—C5	118.3 (3)	C23—C21—C22	107.2 (3)
C7—C6—C5	120.1 (3)	C20—C21—C22	113.4 (3)
C8—C7—C6	118.3 (3)	C23—C21—C3	112.2 (3)
C8—C7—O3	116.6 (3)	C20—C21—C3	99.7 (2)
C6—C7—O3	125.1 (3)	C22—C21—C3	112.3 (3)
C7—C8—C9	122.3 (3)	C21—C22—H22A	109.5
C7—C8—N1	128.2 (3)	C21—C22—H22B	109.5
C9—C8—N1	109.5 (3)	H22A—C22—H22B	109.5
C4—C9—C8	119.0 (3)	C21—C22—H22C	109.5
C4—C9—C3	131.7 (3)	H22A—C22—H22C	109.5
C8—C9—C3	109.3 (3)	H22B—C22—H22C	109.5
C11—C10—C3	107.6 (3)	C21—C23—H23A	109.5
C11—C10—H10A	110.2	C21—C23—H23B	109.5
C3—C10—H10A	110.2	H23A—C23—H23B	109.5
C11—C10—H10B	110.2	C21—C23—H23C	109.5
C3—C10—H10B	110.2	H23A—C23—H23C	109.5
H10A—C10—H10B	108.5	H23B—C23—H23C	109.5
N3—C11—C10	117.5 (3)	C25—C24—O3	129.5 (4)
N3—C11—C12	103.8 (3)	C25—C24—H24	115.2
C10—C11—C12	111.5 (3)	O3—C24—H24	115.2
N3—C11—C20	104.5 (2)	C24—C25—C26	130.5 (4)
C10—C11—C20	104.6 (3)	C24—C25—H25	114.8
C12—C11—C20	115.0 (3)	C26—C25—H25	114.8
N2—C12—C11	108.9 (3)	O2—C26—C25	110.5 (3)
N2—C12—H12A	109.9	O2—C26—C28	109.7 (3)
C11—C12—H12A	109.9	C25—C26—C28	110.9 (3)
N2—C12—H12B	109.9	O2—C26—C27	103.4 (3)
C11—C12—H12B	109.9	C25—C26—C27	110.7 (3)
H12A—C12—H12B	108.3	C28—C26—C27	111.5 (3)
N2—C13—C14	100.2 (3)	C26—C27—H27A	109.5
N2—C13—C19	106.9 (3)	C26—C27—H27B	109.5
C14—C13—C19	116.1 (3)	H27A—C27—H27B	109.5
N2—C13—C18	106.9 (3)	C26—C27—H27C	109.5
C14—C13—C18	115.3 (3)	H27A—C27—H27C	109.5
C19—C13—C18	110.2 (3)	H27B—C27—H27C	109.5
C17—C14—C13	119.7 (3)	C26—C28—H28A	109.5
C17—C14—C15	114.2 (3)	C26—C28—H28B	109.5
C13—C14—C15	102.1 (4)	H28A—C28—H28B	109.5
C17—C14—H14	106.7	C26—C28—H28C	109.5
C13—C14—H14	106.7	H28A—C28—H28C	109.5
C15—C14—H14	106.7	H28B—C28—H28C	109.5
C16—C15—C14	106.0 (3)	N3—C29—H29A	109.5
C16—C15—H15A	110.5	N3—C29—H29B	109.5
C14—C15—H15A	110.5	H29A—C29—H29B	109.5
C16—C15—H15B	110.5	N3—C29—H29C	109.5
C14—C15—H15B	110.5	H29A—C29—H29C	109.5
H15A—C15—H15B	108.7	H29B—C29—H29C	109.5
N2—C16—C15	103.8 (4)		
			
C8—N1—C2—O1	174.9 (3)	C16—N2—C13—C18	73.7 (4)
C8—N1—C2—C3	−5.3 (4)	C12—N2—C13—C18	−55.7 (3)
O1—C2—C3—C9	−173.3 (3)	N2—C13—C14—C17	167.8 (4)
N1—C2—C3—C9	7.0 (3)	C19—C13—C14—C17	−77.6 (5)
O1—C2—C3—C10	−50.0 (4)	C18—C13—C14—C17	53.5 (5)
N1—C2—C3—C10	130.2 (3)	N2—C13—C14—C15	40.5 (4)
O1—C2—C3—C21	65.1 (4)	C19—C13—C14—C15	155.1 (3)
N1—C2—C3—C21	−114.6 (3)	C18—C13—C14—C15	−73.7 (4)
C9—C4—C5—C6	0.3 (5)	C17—C14—C15—C16	−152.3 (4)
C26—O2—C6—C7	−66.8 (4)	C13—C14—C15—C16	−21.5 (5)
C26—O2—C6—C5	116.4 (3)	C12—N2—C16—C15	160.1 (3)
C4—C5—C6—O2	178.3 (3)	C13—N2—C16—C15	33.2 (4)
C4—C5—C6—C7	1.4 (5)	C14—C15—C16—N2	−6.0 (5)
O2—C6—C7—C8	−177.3 (3)	C29—N3—C18—O4	1.6 (5)
C5—C6—C7—C8	−0.5 (5)	C11—N3—C18—O4	−177.4 (3)
O2—C6—C7—O3	−0.9 (5)	C29—N3—C18—C13	−174.5 (3)
C5—C6—C7—O3	175.9 (3)	C11—N3—C18—C13	6.5 (4)
C24—O3—C7—C8	−141.1 (3)	N2—C13—C18—O4	−121.8 (4)
C24—O3—C7—C6	42.5 (5)	C14—C13—C18—O4	−11.4 (5)
C6—C7—C8—C9	−2.2 (5)	C19—C13—C18—O4	122.4 (4)
O3—C7—C8—C9	−178.8 (3)	N2—C13—C18—N3	54.3 (3)
C6—C7—C8—N1	178.3 (3)	C14—C13—C18—N3	164.6 (3)
O3—C7—C8—N1	1.6 (5)	C19—C13—C18—N3	−61.5 (4)
C2—N1—C8—C7	−179.3 (3)	N2—C13—C19—C20	−70.9 (3)
C2—N1—C8—C9	1.1 (4)	C14—C13—C19—C20	178.3 (3)
C5—C4—C9—C8	−2.9 (5)	C18—C13—C19—C20	44.8 (4)
C5—C4—C9—C3	176.9 (3)	C13—C19—C20—C11	17.3 (4)
C7—C8—C9—C4	3.9 (5)	C13—C19—C20—C21	144.4 (3)
N1—C8—C9—C4	−176.5 (3)	N3—C11—C20—C19	−69.8 (3)
C7—C8—C9—C3	−175.9 (3)	C10—C11—C20—C19	166.1 (3)
N1—C8—C9—C3	3.7 (4)	C12—C11—C20—C19	43.4 (4)
C2—C3—C9—C4	173.8 (3)	N3—C11—C20—C21	154.0 (3)
C10—C3—C9—C4	55.7 (5)	C10—C11—C20—C21	29.8 (3)
C21—C3—C9—C4	−67.8 (5)	C12—C11—C20—C21	−92.8 (3)
C2—C3—C9—C8	−6.3 (3)	C19—C20—C21—C23	73.5 (4)
C10—C3—C9—C8	−124.5 (3)	C11—C20—C21—C23	−158.6 (3)
C21—C3—C9—C8	112.1 (3)	C19—C20—C21—C22	−48.1 (4)
C9—C3—C10—C11	−146.2 (3)	C11—C20—C21—C22	79.8 (3)
C2—C3—C10—C11	100.4 (3)	C19—C20—C21—C3	−167.6 (3)
C21—C3—C10—C11	−18.0 (3)	C11—C20—C21—C3	−39.7 (3)
C18—N3—C11—C10	172.4 (3)	C9—C3—C21—C23	−77.2 (4)
C29—N3—C11—C10	−6.6 (5)	C2—C3—C21—C23	35.6 (4)
C18—N3—C11—C12	−64.0 (3)	C10—C3—C21—C23	153.4 (3)
C29—N3—C11—C12	117.0 (4)	C9—C3—C21—C20	163.9 (3)
C18—N3—C11—C20	57.0 (3)	C2—C3—C21—C20	−83.2 (3)
C29—N3—C11—C20	−122.0 (4)	C10—C3—C21—C20	34.5 (3)
C3—C10—C11—N3	−121.9 (3)	C9—C3—C21—C22	43.6 (4)
C3—C10—C11—C12	118.4 (3)	C2—C3—C21—C22	156.5 (3)
C3—C10—C11—C20	−6.5 (3)	C10—C3—C21—C22	−85.8 (3)
C16—N2—C12—C11	−124.8 (3)	C7—O3—C24—C25	−20.7 (6)
C13—N2—C12—C11	−1.1 (4)	O3—C24—C25—C26	−3.5 (7)
N3—C11—C12—N2	59.7 (3)	C6—O2—C26—C25	71.2 (4)
C10—C11—C12—N2	−172.8 (3)	C6—O2—C26—C28	−51.3 (4)
C20—C11—C12—N2	−53.9 (4)	C6—O2—C26—C27	−170.3 (3)
C16—N2—C13—C14	−46.9 (4)	C24—C25—C26—O2	−23.2 (6)
C12—N2—C13—C14	−176.3 (3)	C24—C25—C26—C28	98.6 (5)
C16—N2—C13—C19	−168.3 (3)	C24—C25—C26—C27	−137.2 (5)
C12—N2—C13—C19	62.3 (4)		

### Hydrogen-bond geometry (Å, °)

**Table:**

*D*—H···*A*	*D*—H	H···*A*	*D*···*A*	*D*—H···*A*
N1—H1···O4^i^	0.86	2.21	2.968 (4)	147
C17—H17A···O4	0.96	2.39	3.016 (5)	123
C20—H20···O1	0.98	2.44	3.126 (4)	126

Symmetry codes: (i) −*x*, *y*+1/2, −*z*+1/2.
